# Surface characterization and antibacterial efficiency of well-ordered TiO_2_ nanotube surfaces fabricated on titanium foams

**DOI:** 10.1038/s41598-024-51339-6

**Published:** 2024-01-05

**Authors:** Salih Durdu, Dila Sivlin, Kadriye Ozcan, Selin Kalkan, Ozgul Keles, Metin Usta

**Affiliations:** 1 Department of Industrial Engineering, Engineering Faculty, Giresun University, 28200 Giresun, Turkey; 2 Department of Materials and Metallurgical Engineering, Istanbul Technical University, 34469 Istanbul, Turkey; 3 Department of Genetics and Bioengineering, Giresun University, 28200 Giresun, Turkey; 4 Department of Bioprocess Engineering, Giresun University, 28200 Giresun, Turkey; 5 Department of Materials Science and Engineering, Gebze Technical University, 41400 Gebze/Kocaeli, Turkey; 6 Aluminum Research Center (GTU-AAUM), Gebze Technical University, 41400 Gebze, Turkey

**Keywords:** Biomaterials, Biomaterials

## Abstract

Titanium (Ti)-based implants are not compatible enough due to their bio-inert character, insufficient antibacterial capabilities and stress-shielding problem for dental and orthopaedic implant applications. Thus, this work focused to fabricate, analyze and improve antibacterial properties titanium dioxide (TiO_2_) nanotube array surfaces on Ti foam by anodic oxidation (AO) process. The well-ordered nanotube arrays with approximately 75 nm were successfully fabricated at 40 V for 1 h on Ti foams. Ti and O were observed as major elements on AO-coated Ti foam surfaces. In addition, the existence of TiO_2_ structure was proved on AO-coated foam Ti surfaces. For potential dental and orthopedic implant application, in vitro antibacterial properties were investigated versus *Staphylococcus aureus* and *Escherichia coli*. For both bacteria, antibacterial properties of TiO_2_ nanotube surface were greater than bare Ti foam. The bacterial inhibition versus *Staphylococcus aureus* and *Escherichia coli* of TiO_2_ nanotube surfaces are improved as 53.3% and 69.4% compared to bare Ti foam.

## Introduction

Bone-related problems are commonly observed in clinic applications. Also, these problems economically and socially impose a significant burden. Thereby, due to the many reasons such as aging, the increasing of population and increasing in extreme sports among the young population, bone-related diseases are proposed to increase and become a public health problem^1^. Ti and its alloys, which possess wear resistance, great corrosion resistance and excellent biocompatibility, are the most widely preferred implant materials for dental and orthopaedic defects treatment^2^. However, there are unresolved technical problems on Ti-based implant such as bio-inert character, insufficient antibacterial capabilities and stress-shielding problem^3,4^. Compared to surrounding bone, Ti can lead to problems of stress-shielding due to its relatively high stiffness and this can subsequently result in loosening implant. To overcome stress-shielding problem, Ti foams are investigated for medical implant applications^5–7^. The porosity of Ti foams allows bone ingrowth and interlocking as well as more surfaces for bone-implant contact for medical implant applications^7,8^. The Young’ modulus of the implant, which is close to the human bone, is a vital in decreasing the mechanical mismatch between the implant and bone^9,10^. The low mechanical mismatch avoids stress shielding to the bone and increases the success ratio of the implant^11^. Furthermore, osseointegration is an important problem around Ti-based implant due to bioinert nature of Ti-based implant^12^. Ti cannot chemically bond to bone structure because it is a bioinert structure^5^. This leads to extend healing time. In order to overcome bioinert problem, oxide-based surfaces such as TiO_2_, hydroxyapatite etc. are fabricated on Ti-based implant by various surface modification techniques such as micro arc oxidation^13^, anodic oxidation^14^ etc.

TiO_2_ nanotubes are formed on the Ti sheets/plates by the AO technique^15,16^. The AO technique is a simple and common method to coat well-ordered TiO_2_ nanotubes on Ti substrates^17^. Within last years, TiO_2_ nanotubes arrays have taken huge attentions due to their efficiency in biomaterials since TiO_2_ nanotube surfaces are much more bioactive than Ti substrates^18^. Furthermore, it accelerates the rate of apatite formation and improves bone cell adhesion and proliferation^19,20^. However, very limited investigations were performed on the TiO_2_ nanotubes on Ti foam for implant and other applications in the literature. Huang et al. investigated electrochemical oxidation of carbamazepine in water using enhanced blue TiO_2_ nanotubes on porous Ti foam^21^. Bi et al. examined self-organized amorphous TiO_2_ nanotube on porous Ti foam for rechargeable lithium and sodium ion batteries^22^. Sang et al. evaluated multidimensional anodized titanium foam for solar cell applications^23^. Cao et al. investigated photodegradation properties of TiO_2_ nanotubes on Ti foam^24^. Haghjoo et al. examined the effect of TiO_2_ nanotubes on the biological properties such as cell response of porous Ti foam by AO for orthopaedic applications^25^. Izmir et al. evaluated bioactivity of TiO_2_ nanotubes on Ti6Al4V foams for orthopaedic applications^26^. However, the implant under body conditions is always associated with the risk of bacterial infection. This is caused by the adherence and colonization of bacteria on the surfaces of the implant^27^. The inhibition of bacterial adhesion, proliferation and provision of protection against infection is another goal for implant applications. In view of literature, there is no investigation on antibacterial efficiency of TiO_2_ nanotubes produced on pure Ti foam for medical applications in the literature.

Thus, the aim of this work is to investigate and improve antibacterial ability of TiO_2_ nanotube coated foams against potentially common bacteria (*Staphylococcus aureus* and *Escherichia coli*). In this work, TiO_2_ nanotube surfaces were produced on Ti foam by AO method. Phase structure, surface morphology and elemental structure of TiO_2_ nanotube surfaces on Ti foam were analysed by X-ray diffraction (XRD), scanning electron microscopy (SEM) and energy dispersive spectroscopy (EDX). Importantly, antibacterial efficiencies of TiO_2_ nanotube surfaces on Ti foam were investigated compared to *E. coli* and *S. aureus* for the first time in the literature.

## Experimental details

In this study, in order to produce open foam structure, polyurethane foam (PU) was used as a template. A slurry is prepared using TiH_2_ powder (Alfa Aesar) and polyvinyl alcohol (PVA, Sigma Aldrich), Dolapix, ammonia and distilled water. Dolapix and ammonia were used as dispersants to control viscosity of the slurry and prevent sedimentation. First, distilled water was heated at 95 °C then 32% wt. PVA, 0.8% wt. Dolapix and 1.45% wt. ammonia were added and vigorously mixed by using a magnetic stirrer for 1 h. After the slurry reached homogeneous and transparent state, 60% wt. TiH_2_ powder was slowly added to the slurry under constant stirring. 20 mm × 20 mm × 20 mm size PU foams were immersed in the slurry. By pressing, excess slurry was removed to obtain open cell foam structure as described previous work^6^.

**Figure 1:**
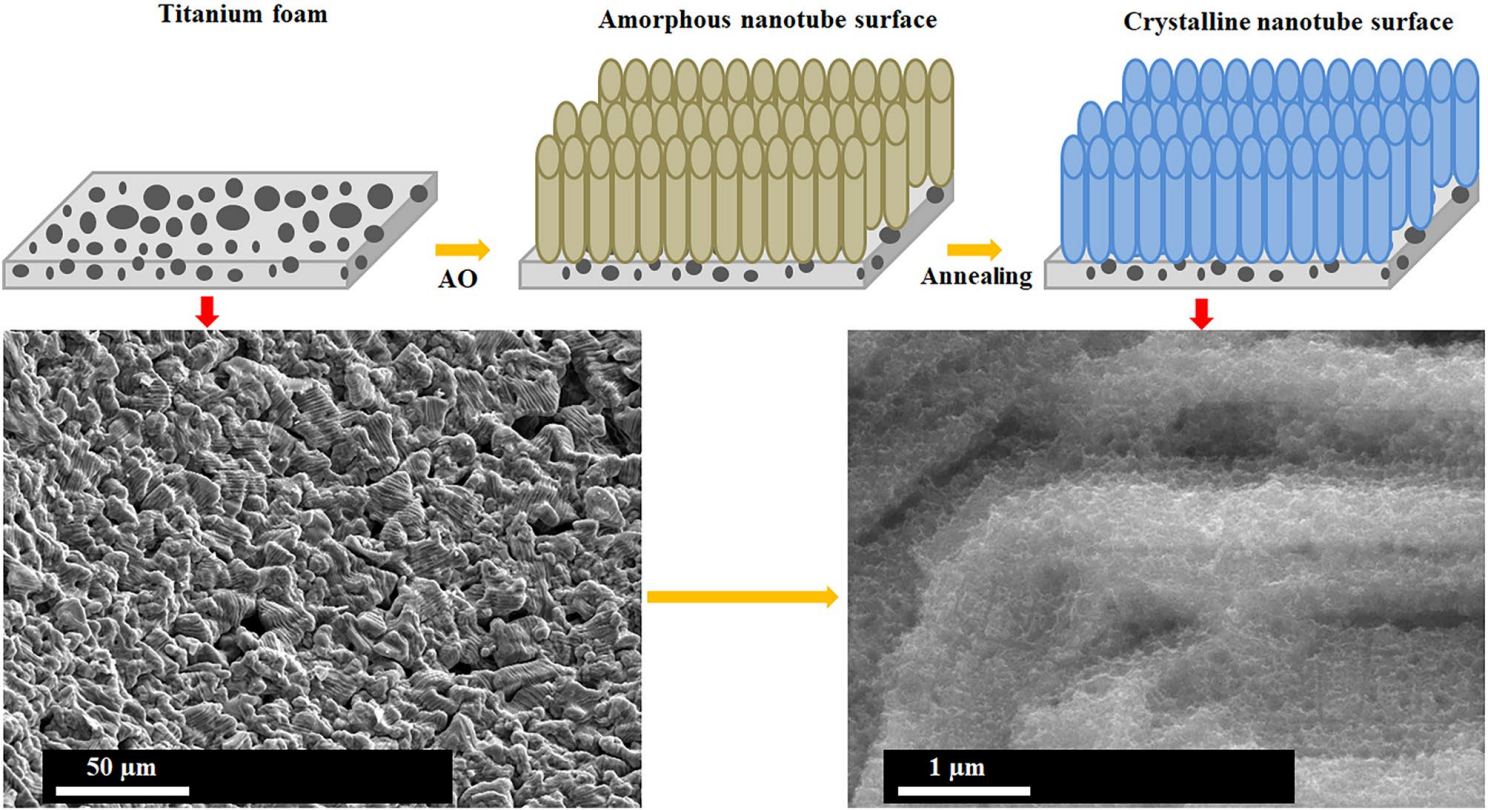
Schematic representation of fabrication of nanotube arrays on Ti foam.

TiO_2_ nanotube coated at 40 V for 60 min in 0.5% wt. NH_4_F and 5.0% vol. distilled water containing ethylene glycol-based solution by a DC power supply (GW-Instek PSU 400). Ti foam and Pt plate were served as an anode and a cathode through AO process. The coated foams were cleaned in distilled water in an ultrasonic bath and they were warmly dried with a heat gun. Due to the amorphous structure of nanotube layers at post-production with AO process, the heat treatment was carried out in a muffle furnace at 450 °C for 60 min without changing the morphology as described previous work^28,29^. Then, they were cooled in the furnace. Schematic representation of fabrication TiO_2_ nanotube arrays on Ti foam by AO process were illustrated in Fig. 1.

The phase structures of the surfaces were analyzed by XRD (Rigaku Dmax 2200) with Cu-Kα radiation at a scanning speed of 1° min^−1^ from 20° to 80°. The surface morphologies of the samples were analyzed by SEM (Philips XL30S FEG). The elemental composition and amounts through the surface were investigated by EDX attached to SEM.

Adhesion of *Staphylococcus aureus* and *Escherichia coli* were evaluated by microbial adhesion experiments. All samples were first sterilized in an autoclave. The test microorganisms were set to a 0.5 McFarland scale and treated with the samples (1 cm^2^) immersed in 5 ml of MHB medium. The samples were then incubated at 37 °C and 125 rpm for 24 h on an orbital shaker. After the samples were removed from the medium, they were washed with 15 ml of water to remove non-adherent organisms, and this procedure was repeated three times. Then, each sample was placed in a clean tube, 2 ml of 150 mM NaCl was added, and shaken for 2 min to collect the bacteria adhering to the surface. Serial dilutions of the collected bacterial solution were prepared and 100 µL of the dilutions were spread on MHA medium. After 48 h of incubation at 37 °C, the number of colonies was measured and the percent inhibition was calculated. All experiments were performed in triplicate.

## Results and discussion

**Figure 2:**
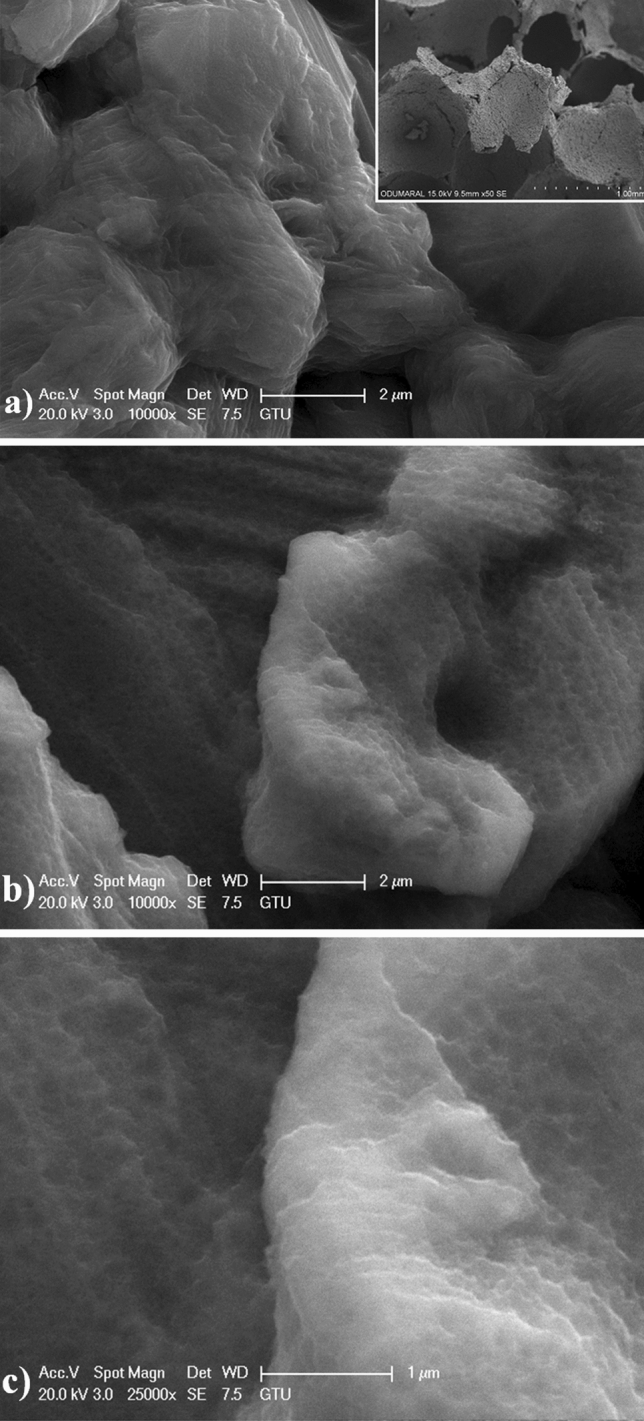
Surface morphologies of the surfaces: (**a**) Ti foam, (**b**) low magnification, and (**c**) high magnification TiO_2_ nanotube layer fabricated on Ti foam.

Surface topographies of Ti foam and nanotube coated foam surfaces were illustrated in Fig. 2. Titanium foams have an open-cell foam structure in which the gas phase dispersed in the matrix is continuous. As a result of the experiments, one-to-one replication of the PU foam was obtained. No significant difference was observed between the samples. However, during the coating of the model material with the mud mixture, it was observed that partially closed cells were formed in some parts of the model material as a result of clogging of the cell walls. The presence of closed cell walls is thought to be due to the high viscosity of the sludge mixture and the excess sludge impregnated with the PU foam cannot be removed by the pressure applied by compressing the foam. The surface of AO coatings consists of well-ordered nanotube arrays with approximately 75 nm through foam structure. The compact TiO_2_ film occurs at the early steps of AO process. Subsequently, this film gradually transforms into a porous layer. The pores randomly grow due to the effective etching of passive film by F^-^ ions within ethylene glycol-based electrolyte. The AO process gradually leads to pores expansion due to long-term field-assisted chemical etching of the AO layer. Furthermore, initial pores develop during the pore rearrangement simultaneously^30,31^. Thus, well-ordered nanotube arrays form on Ti foams.

**Figure 3:**
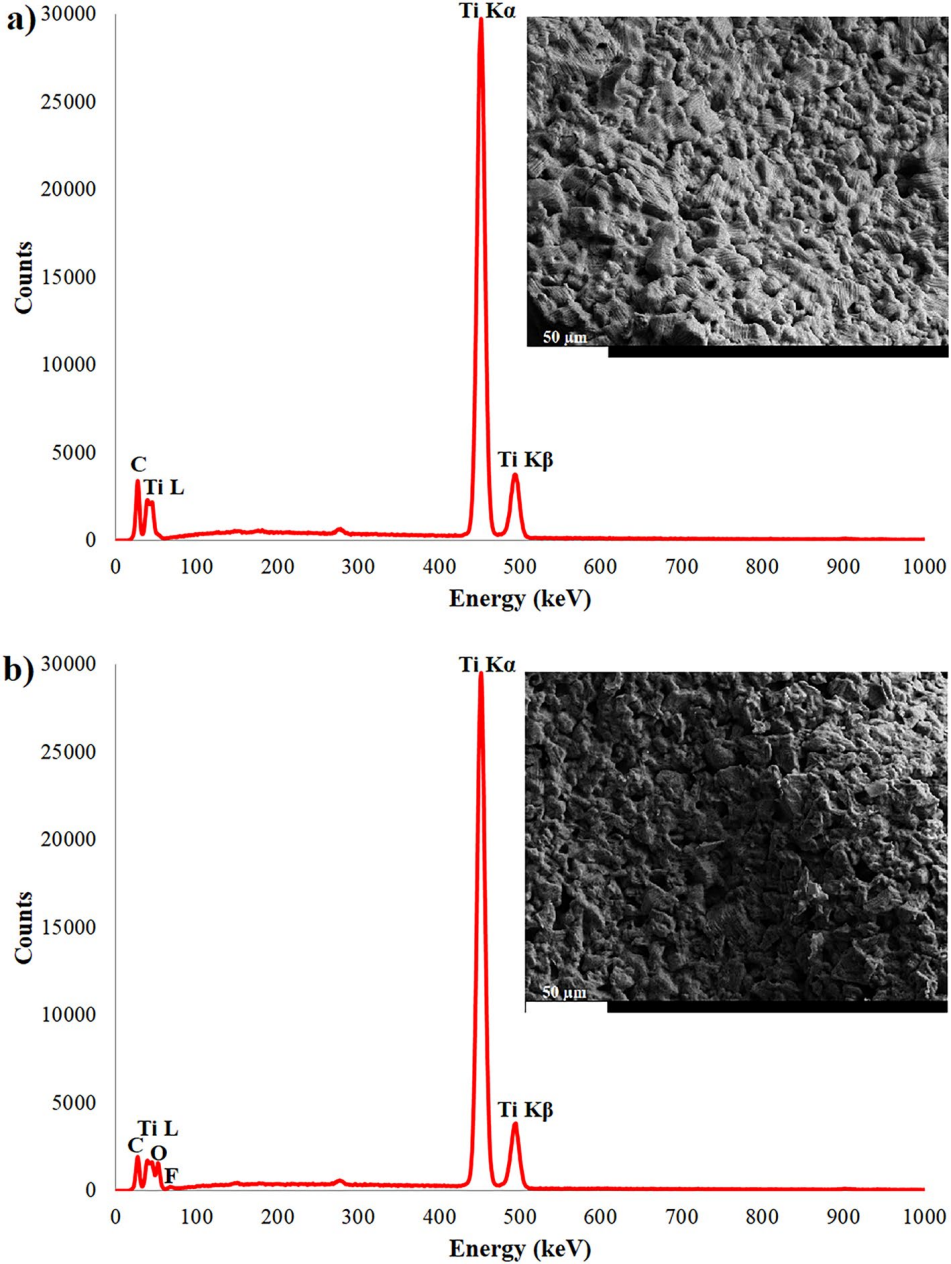
Elemental amount of the surfaces: (**a**) Ti foam and (**b**) AO nanotube layer fabricated on foam.

**Figure 4:**
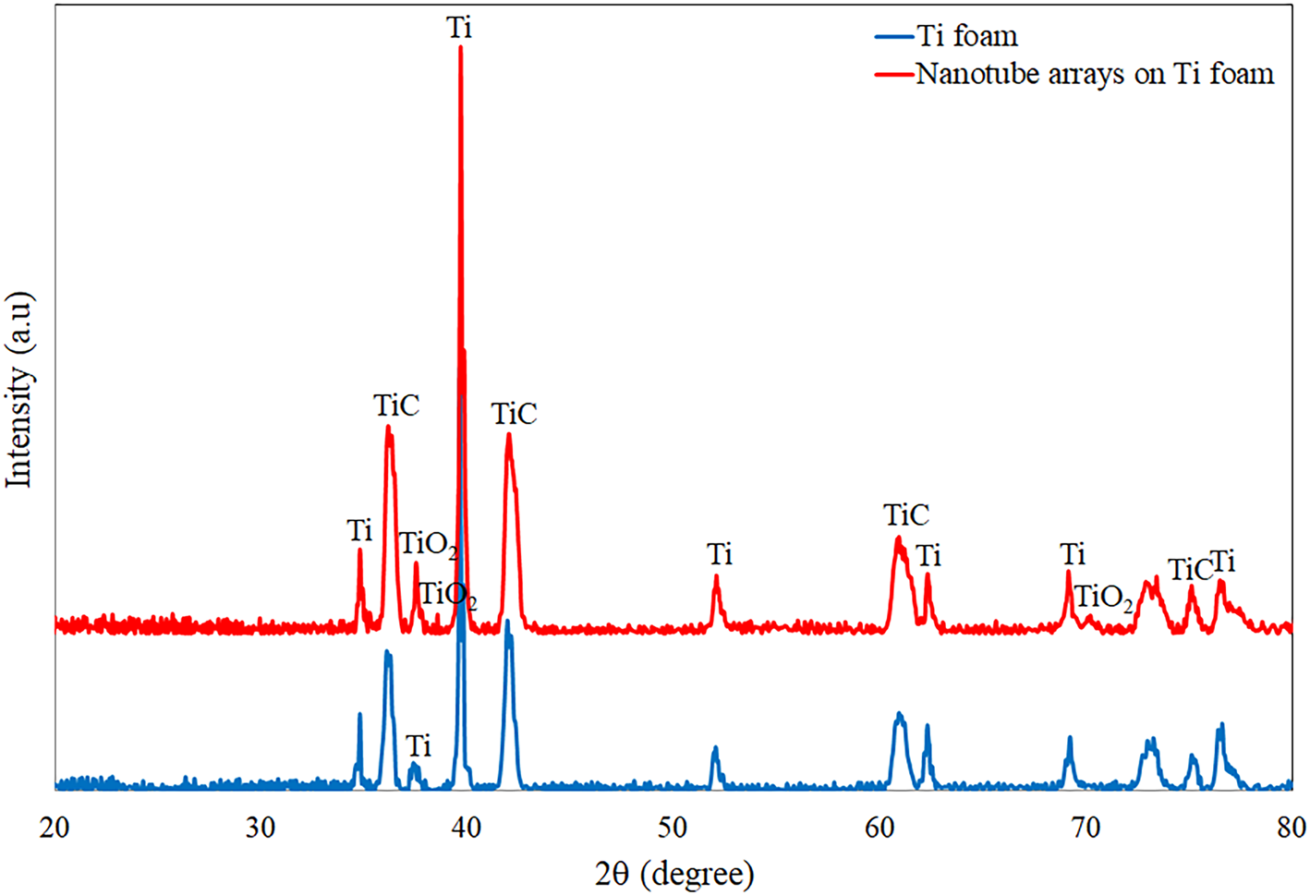
XRD spectra of Ti foam and TiO_2_ nanotube arrays on Ti foam.

Elemental structures of Ti foam and nanotube coated foam surfaces were analyzed by EDX-area as shown in Fig. 3. Only, Ti and C elements were obtained on the surface of uncoated foam (Fig. 3a). The presence of C originates from decomposition of PU foam. The existence of Ti structure comes from foam structure as expected. In addition to Ti and C elements, nanotube coated surface contains both O and F elements as seen in Fig. 3b. The O and F elements exist in nanotube structures. The O and F elements originate in NH_4_F-based electrolyte. Furthermore, the O-based TiO_2_ phase was detected on TiO_2_ nanotube surfaces whereas as shown in Fig. 4. However, there is no the existence of the F-based crystalline phase. Thereby, it could be concluded that the F structure do not form crystalline phases on nanotube surfaces although it presences as the element in the nanotube structures.

Phase structures of bare Ti foam and nanotube arrays fabricated on Ti foams were indicated in Fig. 4. The phase of Ti (# 03-065-3362), TiC (# 00-001-1222) and TiO_2_ (# 00-021-1272) were observed on anodized Ti foam surfaces. It is clear that Ti and TiC come from Ti foam surface. It is reported that TiC is formed by the pyrolysis of binder phenolic resin. The removal temperature of PU foam and the decomposition temperature of the TiH_2_ are nearly identical. Thereby, the C may diffuse interstitial sites forming cubic TiC at post-leaving H_2_ in the system as asserted in the literature^32^. However, the nanotube arrays structure refers to the existence of TiO_2_ at post-heat treatment at 450 °C for 60 min as supported in Fig. 2b, c. The XRD peaks of the annealed nanotube surfaces at 2θ = 37.5°, 38.3° and 70.1° correspond to the (004), (112) and (116) crystallographic orientations of the TiO_2_ (# 00-021-1272). High temperatures such as 500–550 °C could increase the crystallinity of TiO_2_ nanotubes on Ti foam. However, as cited in experimental section, annealing process is applied to the nanotube surfaces on Ti plates 450 °C since morphological differences do not occur on the nanotube surfaces in the many literature studies^14,20,25,28,29,33–37^. If the annealing temperature was applied above 450 °C (500–550 °C), nanotube morphologies are importantly changed and damaged compared to pre-annealing.

**Figure 5:**
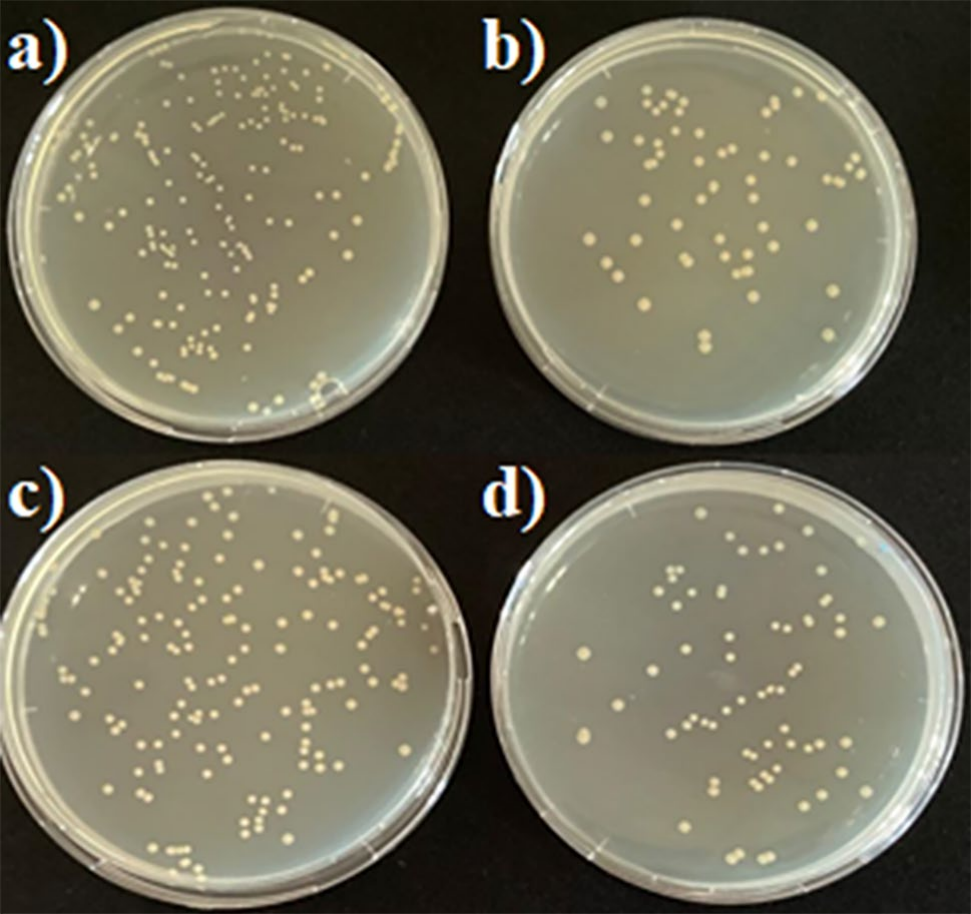
Petri images of bacterial assays: (**a**) Ti foam for *E. coli*, (**b**) TiO_2_ nanotube on Ti foam for *E. coli*, (**c**) Ti foam for *S. aureus* and (**d**) TiO_2_ nanotube on Ti foam for *S. aureus* (All dilutions were performed under identical conditions and petri dishes images indicate identical dilutions).

Table 1 shows the numbers of *E. coli* and *S. aureus* attached to Ti foam and TiO_2_ nanotubes fabricated on Ti foam. Accordingly, TiO_2_ nanotubes doped surfaces decreased the adhesion of both bacteria to the surface compared to Ti foam surfaces. Studies have shown that TiO_2_ has antibacterial properties and has the ability to promote osteogenic differentiation^38^. When the modification of the implant surfaces with TiO_2_ nanostructures is carried out, it will be possible to make the implant more useful in terms of these properties. According to the results, TiO_2_ nanotube doped surfaces were found to be more effective on *E. coli* than *S. aureus* as seen in Fig. 5.

**Table 1:** Bacterial adhesion to the samples and percentage of adhesion inhibition.

Samples	*E. coli*		*S. aureus*	
	CFU 10^4^/ml	Inhibition %	CFU 10^4^/ml	Inhibition %
TiO_2_ nanotubes on Ti foam	0.55 ± 0.004^a^	69.4	0.77 ± 0.005^a^	53.3
Ti foam surface	1.8 ± 0.012^b^		1.65 ± 0.011^b^	

Mean ± standard deviation (n = 3); a, b: means in the same row with different letters differ significantly at p ≤ 0.05.

Many literature studies proposed the possible mechanisms between biological molecules and nanomaterials. It is believed in microbiology that microorganisms and metal oxides carry a negative charge and a positive charge, respectively. Therefore, electromagnetic attraction leads to create between treated surface and the microbe. Bacteria is oxidized at post-contacted the surface and dies instantly^39^. This causes cell inactivation at the signaling levels and regulatory network. Thereby, the respiratory chain’ activity is decreased^40^. All of these depending on the extensive cell wall and the membrane alterations can explain the biocidal activity of TiO_2_ structures such as nanotubes, nanoparticles, etc.

It is known that the antibacterial mechanisms of TiO_2_ including membrane stretching, charge repulsion and surface roughness variation are complex^41^. Charge repulsion between bacteria and TiO_2_ nanotubes prevents the initial adhesion. TiO_2_ possess photocatalytic nature. Thus, one of the main mechanisms of TiO_2_ nanotubes’ action is the generation of reactive oxygen species (ROS) on their surfaces through the photocatalysis process when they were exposed to light at an ideal wavelength^42^. This allows a greater ROS formation. This triggers damage on bacterial cell membrane/DNA etc. eventually, inhibiting the bacteria. Another important antibacterial mechanism of TiO_2_ is membrane stretching. Numerous bacteria such as *E. coli* and *S. aureus* have negative charges on their surfaces. Furthermore, the hydroxyl groups on TiO_2_ nanotube surfaces possess the negative charges. The existence of the same charges between TiO_2_ nanotubes and bacteria occur the repulsive forces reduce bacterial adhesion^43^. Bacteria keep their own shapes due to the difference in osmotic pressure between the inner and outer subshells^44^. Bacteria are initially stretched by the tensile force of the nanotubes and subsequently, it is torn. When the bacteria contacts to TiO_2_ nanotubes, the protruding tube walls increase the surface pressure of bacteria and a part of the membranes suspends over the hollow of the tubes. When the bacteria are consistently adsorbed onto the TiO_2_ nanotubes, the bacteria' surface area is expanded and the suspended membrane stretches further. Thereby, their cell membranes and tissues are damaged. Eventually, the death of the bacteria is accelerated^45,46^. Thus, TiO_2_ nanotube morphologies on Ti foam allowed a greater external and internal contact area with the bacterial solution.

## Conclusions

In summary, antibacterial and bioactive well-ordered TiO_2_ nanotube surfaces were successfully coated on Ti foams by AO technique. For potential dental and orthopedic implant application, in vitro antibacterial properties were investigated versus *S. aureus* and *E. coli*. For *both bacteria*, antibacterial properties of TiO_2_ nanotube surface were greater than bare Ti foam. The bacterial inhibition versus *S. aureus* and *E. coli* of TiO_2_ nanotube surfaces are improved as 53.3% and 69.4% compared to bare Ti foam. Eventually, TiO_2_ nanotube arrays surfaces fabricated on Ti foam significantly possess antibacterial properties under in vitro conditions. So, TiO2 nanotube arrays surfaces fabricated on Ti foam could be potentially candidate for dental and orthopedic implant applications should be investigated in vivo antibacterial and osteogenic activities under in the future.

## Data Availability

The datasets used and/or analyzed during the current study are available from the corresponding author on reasonable request.
